# Aortic remodeling with frozen elephant trunk technique for Stanford type A aortic dissection using Japanese J-graft open stent graft

**DOI:** 10.1007/s00380-018-1246-x

**Published:** 2018-09-06

**Authors:** Masato Tochii, Yoshiyuki Takami, Hiroshi Ishikawa, Michiko Ishida, Yoshiro Higuchi, Yusuke Sakurai, Kentaro Amano, Yasushi Takagi

**Affiliations:** Department of Cardiovascular Surgery, Fujita Health University, 1-98 Dengakugakubo, Kutsukake, Toyoake, Aichi 470-1192 Japan

**Keywords:** Frozen elephant trunk, Aortic dissection, Aortic remodeling

## Abstract

The frozen elephant trunk (FET) technique allows single-stage extended surgical repair of Stanford type A aortic dissection and has shown promotion of aortic remodeling by maintaining the true lumen flow and facilitating its expansion and by promoting false lumen thrombosis. However, few studies have compared the effectiveness of FET technique, in terms of the downstream aortic remodeling. Between 2005 and 2017, 50 patients underwent total arch replacement for Stanford type A aortic dissection, including that with (*n* = 22) and without FET technique (*n* = 28). We compared distal aortic remodeling in patients who underwent total arch replacement with (using a J-Graft open stent graft) or without the technique. The false lumen complete thrombosis rate and the ratio of true lumen area at three levels of the descending aorta were evaluated post operation. In FET group, the diameter and length of the stent graft were 29.0 ± 3.9 mm and 70.9 ± 17.4 mm, respectively. The in-hospital death with and without the FET technique was 0 and 3, respectively, with no late death in both groups. Eight patients (28.6%) only in the non-FET group required additional surgical treatment for downstream aorta. In the FET group, the ratio of true lumen area at the level of bronchial carina and false lumen complete thrombosis rate at the levels of bronchial carina and aortic valve were significantly higher than non-FET group. A more favorable remodeling in the descending aorta was observed in patients who underwent FET associated with a total arch replacement compared to those who underwent total arch replacement alone.

## Introduction

Stanford type A aortic dissection (AAD) is associated with extremely poor prognosis and requires immediate surgical intervention [1, 2]. An emergency operation by a simple ascending aortic replacement may be acceptable, particularly in elderly patients [3]. Aggressive total arch replacement (TAR) is required to avoid a residual dissected aorta with a false lumen patent, particularly in younger patients. Although a previous report demonstrated similar outcomes of conservative and aggressive AAD management, the extent of aortic replacement and prolonged surgical procedure time are considered as significant risk factors of early and late in-hospital death [4–6]. The frozen elephant trunk (FET) technique is a promising surgical approach to promote false lumen obliteration without increasing operative risk [7–12]. It is an important option in the treatment that allows for single-stage repair of complex aortic conditions, such as malperfusion, and provides a landing zone for possible stent graft procedures [10, 13].

The J-Graft open stent graft (JOSG; Japan Lifeline Co., Ltd., Tokyo, Japan), has been available since 2014 in Japan [13]. We started the use of JOSG for only the AAD patients undergoing TAR at our department since 2015 and not for the atherosclerotic thoracic aortic aneurysm patients, owing to the associated paraplegia risk [8, 9]. The FET technique has shown promotion of aortic remodeling by maintaining the true lumen flow and facilitating its expansion and by promoting false lumen thrombosis [7–10, 12, 14, 15]. However, few studies have compared the effectiveness of TAR with and without the FET technique, in terms of the downstream aortic remodeling. We compared downstream aortic remodeling in the AAD patients who had undergone TAR with/without FET technique using JOSG.

## Patients and methods

### Study patients

Between January 2005 and March 2017, 187 patients underwent surgical treatment for AAD at the Department of Cardiovascular Surgery, Fujita Health University, Toyoake, Japan. Of these, 51 patients (27.3%) underwent TAR. Since 2015, we applied the FET technique with JOSG. We compared 22 and 28 patients who underwent TAR with FET using the JOSG (FET group) and without FET (non-FET group), respectively. One patient who underwent FET using a hand-made open stent graft in 2013 was excluded.

### Device and its selection

JOSG comprises a distal stented part, made of a polyester tube, with oval-shaped nitinol stents and a proximal, non-stented graft [13]. It has a unique, interconnected, double-layered, oval-shaped nitinol stents that conform to the aorta curvature. The surgeon selects the graft diameter in increments of 2 mm (21–39 mm); the stented part length can be 60, 90, or 120 mm. The total length of the JOSG is 200 mm.

We diagnosed AAD using enhanced computed tomography (CT). To decide the JOSG size and length, we identified the location of the dissection entry and measured the diameters of the descending aorta in the preoperative CT scans, and we recognized the locations of dissection entry in the scans. The stented part length was decided by measuring the line of the greater curvature from the origin of the left common carotid artery to the descending aorta, without extending to the distal site at the aortic valve level to decrease paraplegia risk. Next, we determined the JOSG diameter to be approximately 90% of the outer aortic diameter or 110% of the true lumen diameter of the descending aorta to use as the distal landing position.

### Surgical strategy and TAR with/without FET

The surgical strategy and technique are mentioned in a previous article [3]. The chest was opened through a median sternotomy under general anesthesia. In most cases, the right axillary artery and right or left femoral artery were exposed for arterial cannulation, with which the cardiopulmonary bypass was performed. The patient temperature was cooled down to 25 °C, followed by lower body circulatory arrest with deep hypothermia. Antegrade selective cerebral perfusion was performed by axillary perfusion with a clamped brachiocephalic artery and direct cannulation of the left common carotid and left subclavian arteries. The operation aimed at excluding the dissection entry. When this entry was located in the ascending aorta, we performed an ascending aortic or hemi-arch replacement, or otherwise, we opted for TAR.

We aggressively opted for FET technique only when the entry was confirmed in the aortic arch during the operation, owing to concerns pertaining to inadvertent insertion of the device tip of the JOSG through the non-identified entry into the false lumen [16]. Therefore, in the non-FET group, we opted for traditional TAR without FET using step-wise technique.

In this group, distal anastomosis was constructed just distal to the left subclavian artery. The aortic arch was transversely resected, and dissecting lumen was reinforced with insertion of BioGlue inside the dissecting lumen. The aortic stump was covered with a paired Teflon felt strip inside and outside the aorta, and the stump was reinforced with continuous 4-0 polypropylene sutures. A synthetic graft with 4 branches was anastomosed end-to-end at the distal aortic arch stump, and antegrade systemic perfusion was initiated. After the proximal anastomosis, the coronary artery was reperfused by aortic declamping. Then left subclavian, left common carotid, and brachiocephalic arteries were anastomosed to the respective graft branches.

In the FET group, distal anastomosis was constructed between the left subclavian and left common carotid arteries. The origin of the left subclavian artery was dissected to close the proximal stump. We performed transesophageal echocardiography proximal to the aortic valve before inducing circulatory arrest using the long-axis view of 3 cm. The JOSG was then inserted and deployed in the true lumen. We adjusted the insertion length based on the non-stented part of the JOSG less than 2 cm, and this part was included in the aortic stump. The subsequent procedures were the same as in the non-FET group.

### Evaluation of distal aortic remodeling after TAR

**Fig. 1:**
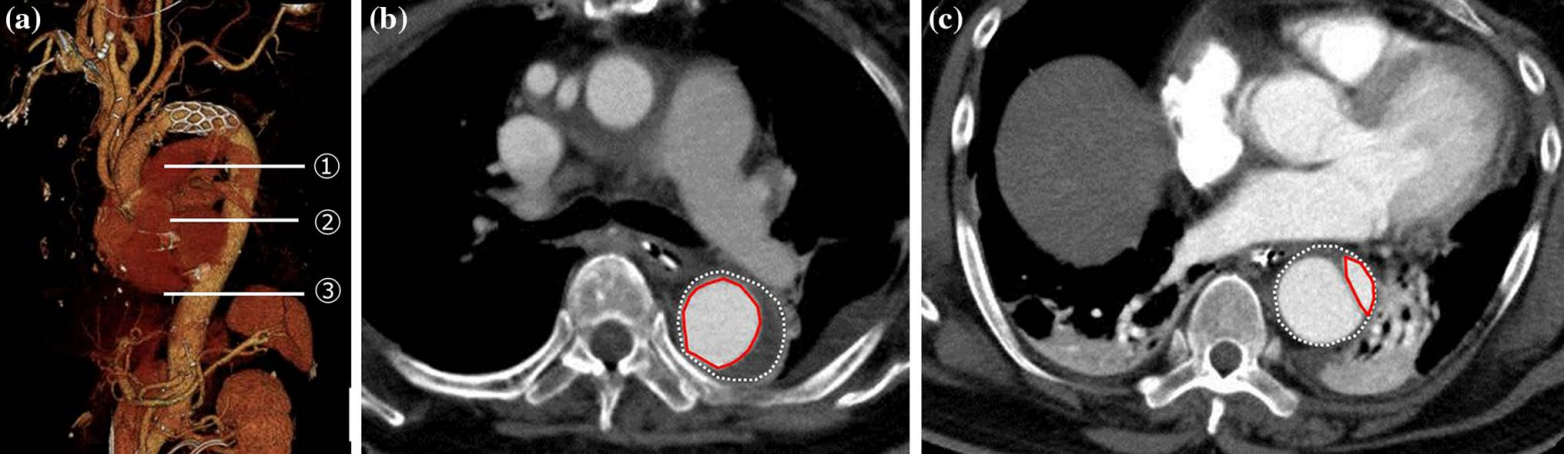
a The evaluated false lumen complete thrombosis rate and the ratio of true lumen area at three levels of bronchial carina (1), aortic valve (2), and diaphragm (3). **b** The false lumen was not enhanced, and therefore defined as “complete thrombosis.” The ratio of true lumen was 55.8%, which was calculated by dividing the traced area of true lumen (red line circle, 750 mm^2^) by the traced area of the whole aorta (dotted line circle, 1350 mm^2^). **c** The false lumen was enhanced, and therefore defined as patent false lumen. The ratio of true lumen was 17.2% (240/1395 mm^2^)

We performed contrast-enhanced CT prior to hospital discharge to evaluate the false lumen complete thrombosis and true lumen expansion rates by measuring the ratio of true lumen area. When the false lumen was not enhanced in the early and delayed phases, it was defined as complete thrombosis. The finding of so-called partial thrombosis of the descending aorta, which manifests as enhancement of false lumen only in the delayed phase, was defined as incomplete thrombosis and patent false lumen. The ratio of true lumen area was calculated at three levels as follows (Fig. 1);

$$ {\text{Ratio of true lumen area }}\left( {\text{\% }} \right) = \frac{\text{Traced area of the true lumen}}{\text{Traced area of the whole aorta  }}   {\text{x }}100 $$

where the area of whole aorta was traced on the adventitia of the descending aorta.

### Data collection and follow-up

We reviewed the clinical records and data of the study patients, including demographics, imaging study results, surgical treatment details, and surgical outcomes. The in-hospital mortality was defined as death caused by any cause during the hospital stay. The follow-up data were obtained, and the institutional review board approved with a waiver of acquiring informed consent.

### Statistical analysis

Continuous variables are expressed as median and interquartile range or mean ± standard deviations. Between-group differences in terms of continuous and categorical variables were assessed with non-parametric (Mann–Whitney) test. Fisher’s *χ*^2^ test was used if the expected frequency was < 5. *P* < 0.05 was considered to be statistically significant. Data were analyzed using StatView 5.0 for Windows (SAS Institute, Inc., Cary, NC, USA).

### Patient demographics and clinical characteristics (Table 1)

In the FET group, all 22 patients underwent TAR with FET technique using JOSG. After introduction of JOSG in 2015, 6 patients were included in the non-FET group treated with traditional TAR.

**Table 1:** Demographic and clinical characteristics of patients

	FET^a^ (*n* = 22)	Non-FET (*n* = 28)	*P*-value
Age, years	57 (39–84)	55 (34–79)	0.7017
Gender, male/female	18/4	18/10	0.2150
Marfan syndrome	2 (9.1)	3 (10.7)	0.6428
Shock	3 (13.6)	0	0.0786
Redo/history of cardiac surgery	0	1 (3.6)	> 0.9999
Coma/loss of consciousness	2 (9.1)	0	0.1886
Organ malperfusion	3 (13.6)	5 (17.9)	> 0.9999

Data presented as *n* (%) or as median (interquartile range)

^a^Frozen elephant trunk

The median age in the two groups was 57 (39–84) years and 55 (34–79) years, for FET and non-FET group, respectively (*p* = 0.7017). The proportion of female patients was comparable in the two groups (18.2% vs. 35.7%, for FET and non-FET group, *p *= 0.2150). Patients with Marfan syndrome and those with a history of previous cardiac surgery were also comparable in the two groups. No significant between-group differences were observed in terms of occurrence of coma or loss of consciousness and frequency of organ malperfusion; preoperative cardiac shock was more frequently recognized in the FET group (13.6% vs. 0%, for FET and non-FET group, *p* = 0.0786).

### Surgical procedures (Table 2)

The rate of concomitant procedures, including aortic root replacement and coronary artery bypass grafting, was comparable in the two groups. The operation and procedure times, including cardiopulmonary bypass, cross-clamp, selective cerebral perfusion, and lower body circulatory arrest time tended to be shorter in the FET group; however, the between-group difference in this respect was not statistically significant. The overall average outer diameter of the JOSG used in this study was 29.0 ± 3.9 mm (range 23–35 mm). The length of the stented part of the JOSG was 60 mm in 15 patients (68.2%), 90 mm in 6 (27.3%), and 120 mm in 1 (4.5%), with an average length of 70.9 ± 17.4 mm.

**Table 2:** Surgical procedures

	FET^a^ (*n* = 22)	Non-FET (*n* = 28)	*P*-*v*alue
Concomitant procedures			
Root replacement	4 (18.2%)	5 (17.9%)	> 0.9999
Coronary artery bypass grafting	1 (4.5)	2 (7.1)	> 0.9999
Operation time, min	592 ± 171 (323–1070)	605 ± 144 (434–1005)	0.7832
Cardiopulmonary bypass time, min	296 ± 80 (179–517)	294 ± 67 (197–474)	0.9500
Cross-clamp time, min	183 ± 49 (99–288)	200 ± 61 (121–359)	0.2976
Selective cerebral perfusion, min	206 ± 57 (76–331)	215 ± 55 (119–340)	0.5742
Lower body circulatory arrest, min	88 ± 24 (46–143)	94 ± 35 (28–226)	0.5286
Stent graft			
External diameter of stent graft			
23 mm	2		
25 mm	5		
27 mm	2		
29 mm	4		
31 mm	3		
33 mm	3		
35 mm	3		
Mean diameter, mm	29.0 ± 3.9		
Length of stent graft			
60 mm	15 (68.2%)		
90 mm	6 (27.3%)		
129 mm	1 (4.5%)		
Mean length, mm	70.9 ± 17.4		

Data presented as *n* (%) or as mean ± standard deviation (range)

^a^Frozen elephant trunk

## Results

### Early and late outcomes (Table 3)

There was no in-hospital deaths in the FET and 3 deaths in the non-FET groups (0% vs. 10.7%, for FET and non-FET group, *p* = 0.2457), respectively. The length of mechanical ventilation after surgery was comparable in the two groups (152 ± 223 h vs. 171 ± 280 h, for FET and non-FET group, respectively; *p* = 0.8319). The stay duration of the surviving patients in the intensive care unit (10.5 ± 11.9 days vs. 12.8 ± 10.4 days, for FET and non-FET group, respectively; *p* = 0.5062) and in the hospital (32.2 ± 20.7 days vs. 49.5 ± 48.2 days, for FET and non-FET group, respectively; *p* = 0.1053) and the major morbidity rates, including re-exploration for bleeding, renal insufficiency requiring hemodialysis, mediastinitis, respiratory failure requiring a tracheotomy, or post-surgical stroke, were comparable in the two groups. Remarkably, none of the patients developed paraplegia.

**Table 3:** Mortality and morbidity

	FET^a^ (*n* = 22)	Non-FET (*n* = 28)	*P*-value
In-hospital mortality	0	3 (10.7)	0.2457
Extubation, hours	152 ± 223 (18–800)	171 ± 280 (20–1344)	0.8319
ICU stay among survivors, days	10.5 ± 11.9 (2–44)	12.8 ± 10.4 (4–49)	0.5062
Hospital stay among survivors, days	32.2 ± 20.7 (13–94)	49.5 ± 48.2 (12–274)	0.1053
Morbidity			
Re-exploration for bleeding	1 (4.5)	2 (7.1)	> 0.9999
Renal insufficiency requiring hemodialysis	2 (9.1)	4 (14.3)	0.6779
Mediastinitis	1 (4.5)	1 (3.6)	> 0.9999
Respiratory failure requiring tracheotomy	3 (13.6)	3 (10.7)	>.9999
Stroke	4 (18.2)	4 (14.3)	0.7155
Paraplegia	0	0	> 0.9999
Additional treatment	0	8 (28.5%)	0.0064
Late death	0	0	> 0.9999

Data presented as *n* (%) or as mean ± standard deviation (range)

*ICU* intensive care unit

^a^Frozen elephant trunk

**Fig. 2:**
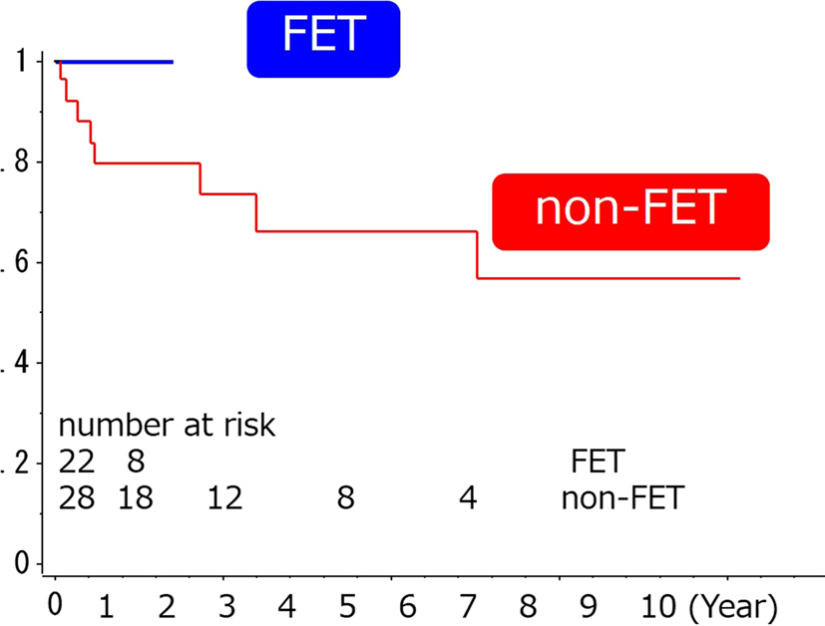
The Kaplan-Meier time-related comparison was used for the freedom from additional surgical treatment after initial TAR in the two groups. The *p* value was not given because of the difference of follow-up duration and short follow-up of FET group, although Log-rank *t*-test was made

There was also no late death in both groups, although there was significant difference in the average follow-up periods [1502 ± 1302 days (range 69–4088) vs. 273 ± 236 days (ranged 53–700), for non-FET and FET group, *p *< 0.0001]. At follow-up, 8 patients in the non-FET group required additional surgical treatment for residual distal aorta, including stent grafting and surgical graft replacement. The average duration from initial TAR to the additional treatment was 659 ± 858 (39–2515) days for non-FET group. Additional surgery included a total of 10 surgical procedures in 8 patients [graft replacement of the descending aorta through left thoracotomy (*n* = 5), endovascular stent grafting for the descending aorta (*n* = 3), and thoracoabdominal aortic replacement (*n* = 2)]. Five of these patients (62.5%) underwent additional surgical procedures within 1 year after the initial emergent TAR, whereas no additional surgical treatment was performed for residual dissecting aorta in the FET group during the follow-up period of 273 ± 236 days (*p *= 0.0064). (Fig. 2).

### False lumen complete thrombosis and ratio of true lumen area (Table 4)

In comparison, the false lumen complete thrombosis rate at the bronchial carina (100% vs. 25%, respectively; for FET and non-FET group, *p *< 0.0001) and aortic valve (72.7% vs. 25%, for FET and non-FET group, respectively; *p* = 0.0329) levels in the FET group was significantly higher. The false lumen thrombosis rate at the diaphragm level showed a higher trend in the FET group (50% vs. 25%, for FET and non-FET group, *p* = 0.3580); however, the difference was not statistically significant.

**Table 4:** False lumen complete thrombosis and ratio of true lumen area

	FET^a^ (*n* = 22)	Non-FET (*n* = 28)	*P*-value
False lumen complete thrombosis at the level of			
Bronchial carina	22 (100)	7 (25)	<0.0001
Aortic valve	16 (72.7)	7 (25)	0.0329
Diaphragm	11 (50.0)	7 (25)	0.3580
Ratio of true lumen area at the level of			
Bronchial carina, %	68.9 ± 18.3	45.9 ± 23.2	0.0008
Aortic valve, %	50.7 ± 25.7	42.0 ± 25.7	0.2355
Diaphragm, %	49.8 ± 26.3	41.8 ± 28.9	0.3522

Data presented as *n* (%) or as mean ± standard deviation (range)

^a^Frozen elephant trunk

**Fig. 3:**
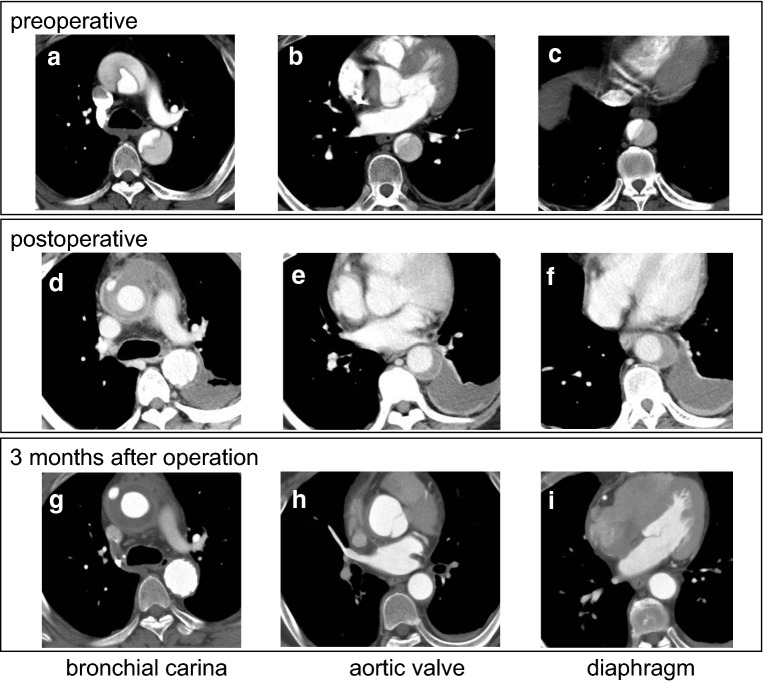
CT scan of a patient treated with TAR with FET. Preoperative CT image (**a**–**c**), postoperative image before discharge from the hospital (**d**–**f**), and at 3 months after the operation (**g**–**i**) at the level of bronchial carina (**a**, **d**, **g**), aortic valve (**b**, **e**, **h**), and diaphragm (**c**, **f**, **i**), respectively. Note excellent aortic remodeling of downstream aorta after TAR with FET with the complete thrombosis of false lumen and expanded true lumen at each level

**Fig. 4:**
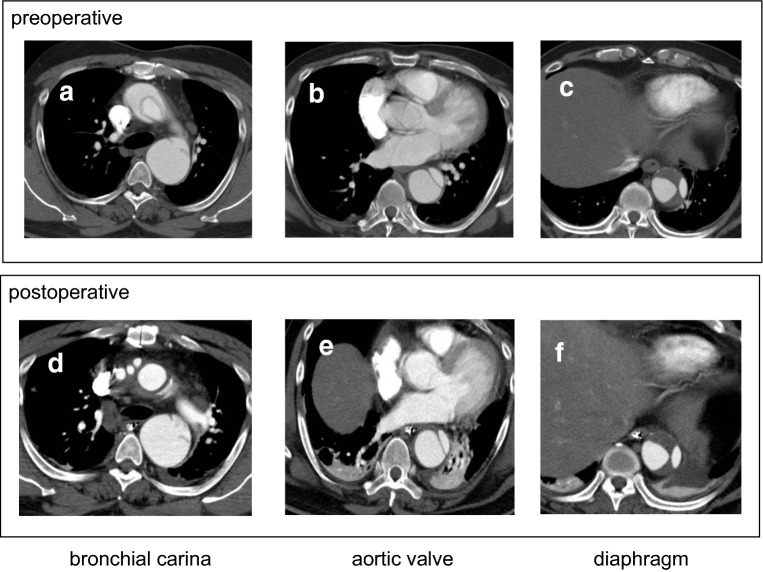
CT scan of a patient treated with TAR without FET before 2015. Preoperative image (**a**–**c**), and postoperative image before discharge from the hospital (**d**–**f**) at the level of bronchial carina (**a**, **d**), aortic valve (**b**, **e**), and diaphragm (**c**, **f**), respectively. Note that, although the elephant trunk was inserted in the descending aorta, the false lumen is patent and the true lumen expansion is not observed at each level of downstream aorta

The ratio of true lumen area at the level of bronchial carina was significantly higher in the FET group compared with that in the non-FET group (68.9 ± 18.3% vs. 45.9 ± 23.2%, for FET and non-FET group, respectively; *p* = 0.0008). The true lumen area ratio in the FET group was greater at the aortic valve (50.7 ± 25.7% vs. 42.0 ± 25.7%, for FET and non-FET group, respectively; *p* = 0.2355) and diaphragm (49.8 ± 26.3% vs. 41.8 ± 28.9%, for FET and non-FET group, respectively; *p* = 0.3522) levels; however, the difference was not statistically significant (Figs. 3 and 4).

## Discussion

Life expectancy and the number of patients who undergo surgery for life-threatening acute AAD have increased over time in Japan. In 2014, in-hospital mortality after approximately 5000 operations for acute AAD was reported to be 10.6% in the Japanese Association for Thoracic Surgery registry [1]. The standard strategy for acute AAD has been ascending aortic replacement, when the primary intimal tear is in the ascending aorta. When the intimal tear is in the distal arch or the descending aorta, TAR is sometimes required as “tear-oriented surgery.” However, extensive graft replacement with TAR is a highly invasive procedure that is associated with an increased mortality and morbidity risk [3–6]. In addition, TAR is associated with a high incidence of false lumen patency of the downstream aorta (range 45.6%–69.7%) [11, 17, 18]. When the false lumen is patent after the initial surgery, the additional surgical procedure rate, which often requires left thoracotomy or re-sternotomy or thoracic endovascular aneurysm repair, ranges from 16% to 26% at 10 years due to descending aortic expansion [11, 18–22]. Some reports demonstrated the feasibility of the FET technique for acute AAD to minimize the incidence of such patency [7, 9–12, 15]. However, few studies compared TAR with/without the FET technique in terms of downstream aortic remodeling.

The main finding of our study is that TAR with FET technique using JOSG was associated with more favorable postoperative distal aortic remodeling compared with that without FET in AAD patients. The favorable aortic remodeling resulted from more expanded true lumen, which eliminates antegrade false lumen flow and promotes complete thrombosis of the false lumen in the descending aorta [7, 10, 14, 21]. Moreover, no additional surgical treatment for residual dissecting aorta was required in the FET group over a follow-up period of 273 ± 236 days; however, five patients in the non-FET group underwent additional surgical procedures within 1 year of the initial emergent TAR. Therefore, TAR with FET technique using JOSG possibly helps to reduce vascular complications associated with dilatation of the residual dissected aorta. In addition, TAR with FET technique using JOSG may save surgical time; in our study, the procedure-related times, including for operation, cardiopulmonary bypass, cardiac ischemia, selective cerebral perfusion, and lower body circulatory arrest, tended to be relatively shorter in the FET group.

To achieve better aortic remodeling, with complete thrombosis of false lumen and true lumen expansion, and to minimize the complication with FET technique, adequate-size JOSG should be selected. We decided the size of the JOSG according to the measurement of the true lumen or the whole aorta based on preoperative CT findings. We determined the JOSG diameter to be about 90% of the outer aortic diameter or 110% of the true lumen diameter of the descending aorta, which we would use as the distal landing position. When the true lumen of the descending aorta was compressed by the false lumen, we selected the stent graft diameter of 90% of the whole aorta, since a previous report demonstrated an 8% increase in the outer diameter due to aortic dissection [8]. The stent part of JOSG comprised a woven structure of Nitinol wire with favorable tractability to the curved aortic arch. However, oversized JOSG may induce intimal damage of descending aorta. Conversely, undersized JOSG may also increase the risk of type 1b endoleak after FET technique, resulting in incomplete thrombosis of the false lumen and inadequate aortic remodeling [23, 24].

The most fatal complication is spinal cord injury (SCI), whose mechanism is believed to be multifactorial, including intraoperative and postoperative blood pressure, distal position of the stent graft, atheromatous emboli of the spinal cord artery, duration of circulatory arrest time and pathology of the aorta [7, 8, 10, 25]. Previous reports demonstrated a higher trend of SCI in atherosclerotic aorta rather than in acute aortic dissection after the FET technique [8, 9]. Therefore, we employ FET technique only for acute aortic dissection.

The length of the JOSG is also an important point for AAD repair; importantly, it excludes the dissection entry in the aortic arch or proximal descending aorta [8–10]. None of the patients developed paraplegia. To decrease paraplegia risk, we avoid excessive insertion of JOSG into the descending aorta as it is unnecessary to insert a long JOSG to achieve aortic remodeling in FET technique that prevents SCI. The most important thing is not to insert the too much long JOSG in the descending aorta. According to our results, 60-mm-length JOSG was effective to achieve the aortic remodeling with low complication rate; however, in some patients, the distal part of the JOSG was placed in the aortic arch. Fortunately, there was no new intimal tear after FET in our series; it is better to place the distal part of JOSG in the descending aorta not in the curved aortic arch. As mentioned, the JOSG length was decided according to the measurement of the greater curvature from the left subclavian artery orifice to the aortic valve level to decrease the paraplegia risk. Paraplegia after the FET technique may be prevented by avoiding deep insertion of the stent graft in the descending aorta. Although some reports reported the possibility of new intimal tear caused by the open stent graft [10, 16, 24] placed in the aortic arch, no patient developed stent graft-induced new entry in our study patients. The very early result of FET is favorable to that of non-FET; however, the long-term result of FET may be favorable or may not be.

There were no significant differences in terms of the false lumen thrombosis rate and the ratio of true lumen area at the level of diaphragm, although beneficial effects were observed at the level of aortic valve and bronchial carina. We believe that the aortic diameter at the diaphragmatic level was affected by two forces: the radial force of blood through the true lumen maintained by the JOSG and the false lumen pressure from more distal re-entry blood flow. The existence of re-entries in the mid-descending aorta distal to the end of FET, those around the celiac trunk or superior mesenteric artery might influence the patency of the false lumen in the descending aorta. And whether FET excluded the re-entry in the proximal descending aorta may also influence the proximal false lumen patency. Long-term follow-up is required to observe the potential adverse effects of abdominal re-entry and residual dissection, since residual dissection with a patent false lumen was shown to decrease survival rate and increase aortic events of the downstream aorta [19].

The Kaplan-Meier time-related comparison was used for the freedom from additional surgical treatment after initial TAR in the two groups. The p value was not given because of the difference of follow-up duration and short follow-up of FET group, although Log-rank *t* test was made. However, no patient underwent additional surgical treatment for downstream aorta in FET group, although 8 of 28 patients (28.5%) underwent additional surgical treatment for downstream aorta in non-FET group during follow-up period. Of those, 5 of 8 patients (62.5%) underwent additional surgical treatment within 1 year after initial TAR in non-FET group, although no patient required those procedures in FET group within 1 year after the initial TAR. It was suggested that the FET procedure can reduce the additional surgical treatment for residual aorta.

In conclusion, TAR with FET technique using JOSG resulted in more favorable postoperative aortic remodeling compared with that without FET in AAD patients. This favorable aortic remodeling resulted from more expanded true lumen, which eliminates antegrade false lumen flow and promotes complete thrombosis of the false lumen in the descending aorta. These favorable effects are expected to contribute to freedom from long-term complications associated with dilatation of the residual dissected aorta.

## Limitations

The retrospective study design, single-center scope, and relatively small sample size are key limitations of the study. The patient categorization was not random (FET and non-FET groups) and the treatment strategy was based on the patient’s condition and surgeon’s preference. Lastly, the follow-up duration was shorter in the FET group. Although no additional surgical treatment for downstream aorta was performed in this group, longer observation is required to confirm the benefits of aortic remodeling associated with this technique.
