# Correction to: Aortic remodeling with frozen elephant trunk technique for Stanford type A aortic dissection using Japanese J-graft open stent graft

**DOI:** 10.1007/s00380-018-1268-4

**Published:** 2018-09-26

**Authors:** Masato Tochii, Yoshiyuki Takami, Hiroshi Ishikawa, Michiko Ishida, Yoshiro Higuchi, Yusuke Sakurai, Kentaro Amano, Yasushi Takagi

**Affiliations:** Department of Cardiovascular Surgery, Fujita Health University, 1‑98 Dengakugakubo, Kutsukake, Toyoake, Aichi 470-1192 Japan

## Correction to: Heart and Vessels 10.1007/s00380-018-1246-x

The article Aortic remodeling with frozen elephant trunk technique for Stanford type A aortic dissection using Japanese J-graft open stent graft, written by Masato Tochii, Yoshiyuki Takami, Hiroshi Ishikawa, Michiko Ishida, Yoshiro Higuchi, Yusuke Sakurai, Kentaro Amano and Yasushi Takagi was originally published electronically on the publisher’s internet portal (currently SpringerLink) on 06 September 2018 without open access.

With the author(s)’ decision to opt for Open Choice the copyright of the article changed on 26 September 2018 to © The Author(s) 2018 and the article is forthwith distributed under the terms of the Creative Commons Attribution 4.0 International License (http://creativecommons.org/licenses/by/4.0/), which permits use, duplication, adaptation, distribution and reproduction in any medium or format, as long as you give appropriate credit to the original author(s) and the source, provide a link to the Creative Commons license and indicate if changes were made.

In addition, the Table 2 was published incorrectly in the original publication of the article and the correct Table 2 is provided.

**Table 2:** Surgical procedures

	FET^a^ (*n* = 22)	Non-FET (*n* = 28)	*P* value
Concomitant procedures			
Root replacement	4 (18.2%)	5 (17.9%)	> 0.9999
Coronary artery bypass grafting	1 (4.5)	2 (7.1)	> 0.9999
Operation time, min	592 ± 171 (323–1070)	605 ± 144 (434–1005)	0.7832
Cardiopulmonary bypass time, min	296 ± 80 (179–517)	294 ± 67 (197–474)	0.9500
Cross-clamp time, min	183 ± 49 (99–288)	200 ± 61 (121–359)	0.2976
Selective cerebral perfusion, min	206 ± 57 (76–331)	215 ± 55 (119–340)	0.5742
Lower body circulatory arrest, min	88 ± 24 (46–143)	94 ± 35 (28–226)	0.5286
Stent graft			
External diameter of stent graft			
23 mm	2		
25 mm	5		
27 mm	2		
29 mm	4		
31 mm	3		
33 mm	3		
35 mm	3		
Mean diameter, mm	29.0 ± 3.9		
Length of stent graft			
60 mm	15 (68.2%)		
90 mm	6 (27.3%)		
120 mm	1 (4.5%)		
Mean length, mm	70.9 ± 17.4		

Data presented as *n* (%) or as mean ± standard deviation (range)

^a^Frozen elephant trunk

